# Targeting lipoprotein(a): Pharmacotherapy in focus

**DOI:** 10.1093/ehjcvp/pvae102

**Published:** 2025-01-02

**Authors:** Doreen Su-Yin Tan, Zi Heng Ooi, Claire Sook Fui Lew

**Affiliations:** Department of Pharmacy and Pharmaceutical Sciences, National University of Singapore, Block S4A, Level 3, 18 Science Drive 4, Singapore, 117543; Department of Cardiology, National University Heart Centre, National University Hospital, 5 Lower Kent Ridge Road, Singapore, 119074; Department of Pharmacy and Pharmaceutical Sciences, National University of Singapore, Block S4A, Level 3, 18 Science Drive 4, Singapore, 117543; Department of Pharmacy, Tan Tock Seng Hospital, 11 Jalan Tan Tock Seng, Singapore, 308433

High lipoprotein(a) [Lp(a)] levels is an independent risk factor for the development of atherosclerotic cardiovascular diseases (ASCVD) and aortic stenosis. It is believed to contribute to residual ASCVD risk in patients at all levels of low-density lipoprotein (LDL) control.^1,2^ Pathophysiology of Lp(a)-associated ASCVD has been characterized in many studies with Lp(a) being considered as pro-atherosclerotic, pro-thrombotic, anti-fibrinolytic, and inflammatory.^2,3^ Lp(a) is largely genetically determined and remains relatively unchanged in a person's life; with lifestyle management having minimal impact on its levels.^1,4^ Interestingly, some small studies showed some Lp(a) reduction with nutraceuticals use; however, its effectiveness is still debatable.^3^ Presently, lipoprotein apheresis is the only approved treatment for lowering Lp(a), but its use is limited because it is time- and resource-intensive for what could be considered modest reduction in Lp(a).^1,5^ Current lipid-lowering pharmacotherapies have limited effectiveness and there are no approved agents directed solely at lowering Lp(a). Nevertheless, pharmacological approaches have been explored, with novel agents under investigation.^1,2,4,5^

Lipid-lowering therapies Lp(a) effects on are summarized in *Table 1*. Statins play a well-defined role in reducing LDL and ASCVD risk. Despite structural similarities with LDL, statins *are* not *effective in* -only not- *lowering* Lp(a) substantially; *in fact*, some studies suggest slight increase in Lp(a) associated with statin use.^1^ The mechanism behind the increase is unclear, but it is unlikely to have an impact on ASCVD risk due to the established cardiovascular benefits of statins.^1,4,5^ The Lp(a) lowering effects of ezetimibe are poorly understood, with most evidence suggesting minimal effect.^1,3,4^ Other lipid-lowering agents like, bile acid sequestrants, fibrates, and bempedoic acid, do not lower Lp(a); instead, they could cause a modest increase.^1^ Niacin, in contrast, has been shown to reduce Lp(a) by 20–40%. However, the lack of established cardiovascular benefit and undesirable side effects poses a challenge to its use for Lp(a) reduction.^1–3^ Proprotein convertase subtilisin/kexin type 9 (PCSK9) inhibitors, have been shown to significantly lower LDL by 50–60% as monotherapy or as add-on to statins.^4^ Alirocumab and evolocumab showed significant Lp(a) reduction at −23 and −26.9%, respectively. Furthermore, post-hoc analysis suggests these Lp(a) reductions may contribute to cardiovascular risk reduction, independent of LDL reduction.^3,4^ Inclisiran, a small interfering RNA of PCSK9, showed reduction in Lp(a) by up to 26% but its cardiovascular benefits are still under investigation.^1,3^ Newer similar agents aimed at reducing Lp(a) such as olpasiran, lepodisiran, and zerlasiran have all demonstrated more than 95% Lp(a) reduction in early phase trials. Agents used in familial hypercholesterolaemia; evinacumab, lomitapide, and mipomersen have also demonstrated reduction in Lp(a) (*Table 1*).^1,3^ A novel antisense oligonucleotide, Pelacarsen—designed specifically to reduce Lp(a) by interfering with Lp(a) synthesis, demonstrated a promising over 80% reduction in Lp(a) levels.^3–5^ With the cardiovascular outcome trials underway for many of these newer agents, there are new and promising ways for new approaches in the management of elevated Lp(a). Until the new agents are in clinical use, the best choices are likely to be Niacin or the PCSK9-based treatments.

**Table 1:** Effect of lipid-lowering therapies on Lp(a) levels

Lipid-lowering therapy		Effect on Lp(a) levels
**Current therapies associated with decrease in Lp(a)**		
Niacin		−20 to −40%^3^
PCSK9 inhibitors	Evolocumab	−26.9% (IQR, −6.2 to −46.7%)^1^
	Alirocumab	−23% (IQR, −47 to 0%)^1^
Small interfering RNA targeting PCSK9	Inclisiran	−18.6 to −26%^3^
**Newer therapies in the pipeline associated with decrease in Lp(a)**		
Small interfering RNA targeting PCSK9	Zerlasiran	−98%^4^
	Lepodisiran	−97%^3^
	Olpasiran	−71 to −98%^3^
Pelacarsen		−66 to −92%^3^
Mipomersen		−26.4%^3^
Lomitapide		−17%^3^
Evinacumab		−5.5%^1^
**Current therapies associated with increase in Lp(a)**		
Bile acid sequestrants		20%^1^
Fibric acid derivatives		1.6–20%^1^
Statins		8.5–19.6%^1^
Bempedoic acid		2.4%^1^
Ezetimibe		Minimal effect^1,3^

Abbreviations: Lp(a), lipoprotein(a); PCSK9, proprotein convertase subtilisin/kexin type 9.

## References

[1] Alhomoud IS, Talasaz A, Mehta A, Kelly MS, Sisson EM, Bucheit JD. Role of lipoprotein(a) in atherosclerotic cardiovascular disease: a review of current and emerging therapies. Pharmacotherapy 2023;43:1051–1063. 10.1002/phar.2851 37464942

[2] Reyes-Soffer G, Ginsberg HN, Berglund L, Duell PB, Heffron SP, Kamstrup PR. Lipoprotein(a): a genetically determined, causal, and prevalent risk factor for atherosclerotic cardiovascular disease: a scientific statement from the American Heart Association. Arterioscler Thromb Vasc Biol 2022;42:e48–e60. 10.1161/ATV.0000000000000147 34647487 PMC9989949

[3] Baragetti A, Da Dalt L, Norata GD. New insights into the therapeutic options to lower lipoprotein(a). Eur J Clin Investigation 2024;54:e14254. 10.1111/eci.14254 38778431

[4] Nordestgaard BG, Langsted A. Lipoprotein(a) and cardiovascular disease. The Lancet 2024;404:1255–1264. 10.1016/S0140-6736(24)01308-4 39278229

[5] Nicholls SJ . Therapeutic Potential of lipoprotein(a) inhibitors. Drugs 2024;84:637–643. 10.1007/s40265-024-02046-z 38849700 PMC11196316

